# Can Botulinum Toxin Type E Serve as a Novel Therapeutic Target for Managing Chronic Orofacial Pain?

**DOI:** 10.3390/toxins17030130

**Published:** 2025-03-10

**Authors:** Sung-Koog Jung, Yu-Mi Kim, Min-Jeong Jo, Jo-Young Son, Jin-Sook Ju, Min-Kyoung Park, Min-Kyung Lee, Jae-Young Kim, Jeong-Sun Nam, Dong-Kuk Ahn

**Affiliations:** 1 Department of Oral Physiology, School of Dentistry, Kyungpook National University, Daegu 41940, Republic of Korea; ln3000@hanmail.net (S.-K.J.); boboatom@naver.com (Y.-M.K.); dmvvv3@naver.com (M.-J.J.); n-violetjy@nate.com (J.-Y.S.); jsju@knu.ac.kr (J.-S.J.); 2 Department of Dental Hygiene, Kyung-Woon University, Gumi 39160, Republic of Korea; bukimin@hanmail.net; 3 Department of Dental Hygiene, Dong-Eui University, Busan 47340, Republic of Korea; lmk849@deu.ac.kr; 4 JETEMA, Global 225-12, Pangyoyeok-ro, Bundang-gu, Seongnam 13494, Republic of Korea; jykim@jetema.com (J.-Y.K.); jsnam@jetema.com (J.-S.N.)

**Keywords:** botulinum toxin type E, antinociception, formalin, complete Freund’s adjuvant, trigeminal neuropathic pain

## Abstract

The existing literature offers limited experimental evidence on the role of botulinum neurotoxin type E (BoNT-E) in pain transmission. The present study investigated the antinociceptive effects of subcutaneously administered BoNT-E in chronic orofacial pain conditions. This study used orofacial formalin-induced pronociceptive behavior and complete Freund’s adjuvant (CFA)-induced thermal hyperalgesia as inflammatory pain models in male Sprague Dawley rats. A neuropathic pain model was also developed by causing an injury to the inferior alveolar nerve. Subcutaneously administered BoNT-E (6, 10 units/kg) significantly reduced nociceptive behavior during the second phase of the formalin test compared to that of the vehicle treatment. These doses similarly alleviated thermal hypersensitivity in the CFA-treated rats. Moreover, BoNT-E (6, 10 units/kg) markedly attenuated mechanical allodynia in rats with an inferior alveolar nerve injury. At a dose of 10 units/kg, BoNT-E produced antinociceptive effects that became evident 8 h post-injection and persisted for 48 h. Notably, BoNT-E (10 units/kg) significantly reduced the number of *c-fos*-immunostained neurons in the trigeminal subnucleus caudalis of rats with an inferior alveolar nerve injury. In comparison, intraperitoneally administered gabapentin (30, 100 mg/kg) demonstrated significant mechanical anti-allodynic effects but exhibited lower analgesic efficacy than that of BoNT-E. These findings highlight the potential of BoNT-E as a therapeutic agent for chronic pain management.

## 1. Introduction

Botulinum neurotoxins (BoNTs), produced by the anaerobic bacterium *Clostridium botulinum*, is an exceptionally potent neurotoxic protein comprising heavy and light chains that are interconnected by disulfide bonds [1,2,3,4]. BoNTs are primarily known for their ability to inhibit acetylcholine release by disrupting the soluble N-ethylmaleimide-sensitive factor attachment receptor (SNARE) protein complex at peripheral motor nerve terminals [4,5]. There are seven principal types of BoNTs, classified as types A through G, with types A, B, and E serving as the primary etiological agents of botulism in humans [3,6,7].

Recently, BoNT type A (BoNT-A) has been widely used in aesthetics and therapeutic fields, leveraging its toxic properties for beneficial outcomes [8,9]. While the primary mechanism of BoNT-A involves the blockade of acetylcholine release at the neuromuscular junction, resulting in muscle paralysis, numerous experimental studies have indicated its efficacy in managing chronic persistent pain. BoNT-A has shown effectiveness in alleviating inflammatory pain induced by carrageenan [10], formalin [11,12], and complete Freund’s adjuvant (CFA) [12]. Furthermore, BoNT-A has been demonstrated to reduce neuropathic pain resulting from a partial sciatic nerve transection [13], spinal nerve ligation at L5/L6 [14], chronic sciatic nerve constriction [15], inferior alveolar nerve injury [16], and trigeminal nerve root compression [17].

BoNT type E (BoNT-E) inhibits SNARE-mediated synaptic vesicle fusion by cleaving synaptosomal-associated protein 25 (SNAP-25) [4,18,19]. Several studies have demonstrated that BoNT-E exhibits long-term neuroprotective properties, including reducing the loss of hippocampal pyramidal neurons in experimental stroke models [20]. Furthermore, BoNT-E has been shown to decrease the chronic seizure frequency in a mouse epilepsy model by inhibiting glutamate release and suppressing pyramidal neuron spiking activity [21,22]. These findings suggest that the hippocampal administration of BoNT-E might provide therapeutic benefits, offering antidotal and antiepileptogenic effects in experimental epilepsy models [23]. A recent clinical trial suggested that a single dose of BoNT-E produced modest improvements in itch and pain conditions following excisional repairs on the forehead compared to a placebo [24]. Although a previous study in humans reported that BoNT-E attenuates pain, there is limited experimental evidence in laboratory animals concerning its role in pain transmission. Therefore, the precise role of BoNT-E in pain transmission must be clarified.

This study investigated the antinociceptive effects of subcutaneously administered BoNT-E in orofacial inflammatory pain models. Behavioral responses were elicited via formalin and CFA injections into the facial region to induce inflammatory pain in rats. Additionally, the impact of BoNT-E on mechanical allodynia was assessed in rats with inferior alveolar nerve injuries. To confirm the antinociceptive effects of BoNT-E, changes in *c-fos* expression within the trigeminal subnucleus caudalis were evaluated following subcutaneous BoNT-E administration.

## 2. Results

### 2.1. Changes in Motor Performance and Body Weights

**Figure 1:**
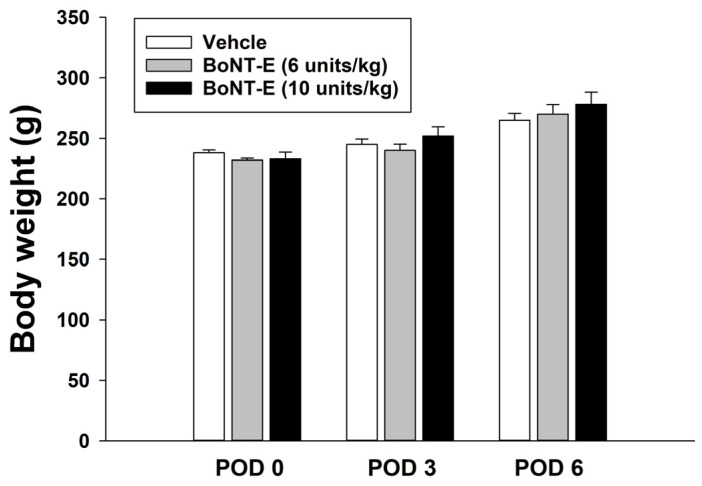
Changes in animal body weight following inferior alveolar nerve injury. BoNT-E was administered on post-operative day 3 (POD 3). The administration of BoNT-E did not significantly affect body weight compared to the vehicle treatment.

The present study evaluated motor impairment following the subcutaneous injection of BoNT-E in the hind leg. The administration of BoNT-E (10 units/kg) resulted in a latency time of 98 ± 17 s, which did not differ significantly from that of the vehicle injection (84 ± 15 s), as demonstrated by the normal performance in the rotarod test. Additionally, this study assessed changes in body weight following BoNT-E injection in rats with an inferior alveolar nerve injury. BoNT-E administration did not significantly affect body weight compared to the vehicle treatment (Figure 1).

### 2.2. Subcutaneously Injected BoNT-E-Attenuated Formalin-Induced Nociceptive Behavior

**Figure 2:**
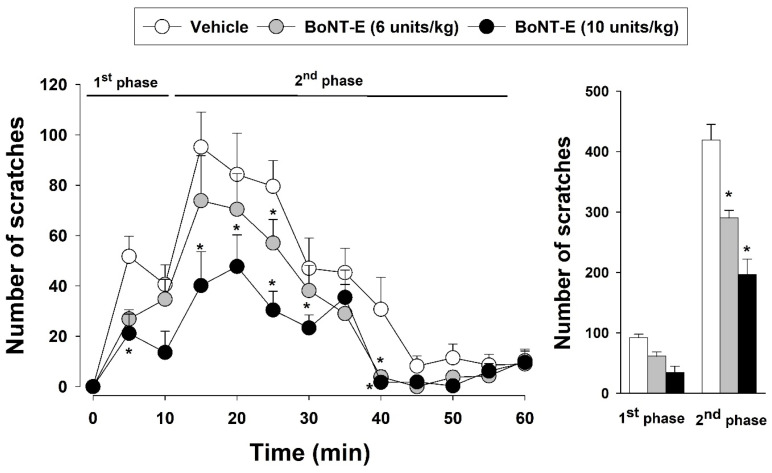
Changes in formalin-induced nociceptive behavior after botulinum neurotoxin type E (BoNT-E) treatment. Administration of 5% formalin resulted in biphasic nociceptive behavior. Subcutaneous pretreatment with BoNT-E (6, 10 units/kg) 8 h prior to injecting 5% formalin significantly reduced nociceptive behavior during the second phase. Each group comprised seven animals. * *p* < 0.05, BoNT-E-treated vs. vehicle-treated group.

The effects of BoNT-E on the orofacial pronociceptive responses induced by formalin are illustrated in Figure 2. The subcutaneous administration of 5% formalin (30 μL) triggered pronounced pronociceptive behaviors. However, BoNT-E that was administered subcutaneously at doses of 6 and 10 units/kg significantly attenuated these formalin-induced pronociceptive behaviors compared those of to the vehicle treatment group (F_(2,18)_ = 355.926, *p* < 0.05). Additionally, this study examined the influence of BoNT-E on the biphasic nociceptive response elicited by formalin. BoNT-E administration (6, 10 units/kg) significantly reduced nociceptive scratching behavior during the second phase compared to that of the vehicle treatment (*p* < 0.05).

### 2.3. Subcutaneously Injected BoNT-E-Attenuated CFA-Induced Thermal Hyperalgesia

**Figure 3:**
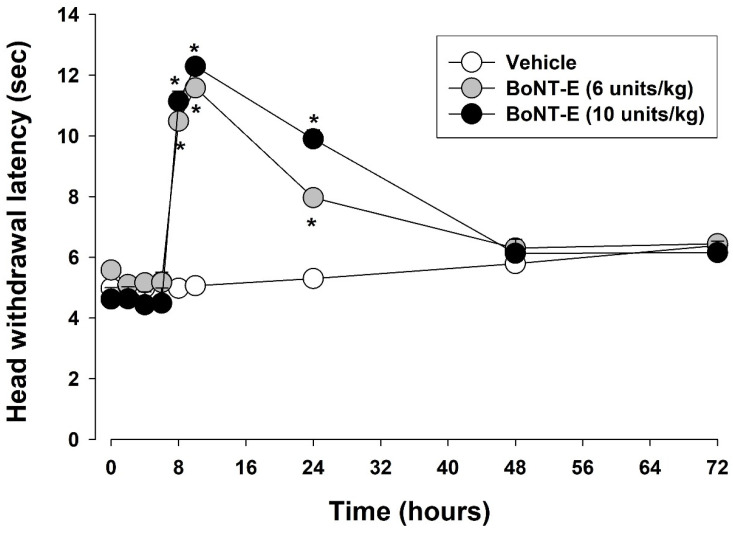
Changes in head withdrawal latency following subcutaneously administered botulinum neurotoxin type E (BoNT-E) in rats treated with complete Freund’s adjuvant (CFA). Subcutaneous injection of CFA induced thermal hyperalgesia within 1 day after injection, which persisted for 10 days. BoNT-E treatment (6, 10 units/kg) at 3 days significantly alleviated thermal hyperalgesia. Each group comprised seven animals. * *p* < 0.05, BoNT-E-treated vs. vehicle-treated group.

Figure 3 illustrates the antinociceptive effects of BoNT-E on thermal hyperalgesia induced by CFA injection in the orofacial region. Three days after the CFA injection, the subcutaneous administration of BoNT-E at doses of 6 or 10 units/kg significantly increased the head withdrawal latency in CFA-treated rats (F_(2,18)_ = 3.241, *p* < 0.05). The antinociceptive effects were detectable 8 h post-injection and persisted for 24 h following the 10 units/kg of BoNT-E injection. In contrast, the subcutaneous vehicle injection did not elicit any significant changes in the head withdrawal latency in the CFA-treated rats.

### 2.4. Subcutaneously Injected BoNT-E-Attenuated Neuropathic Mechanical Allodynia

**Figure 4:**
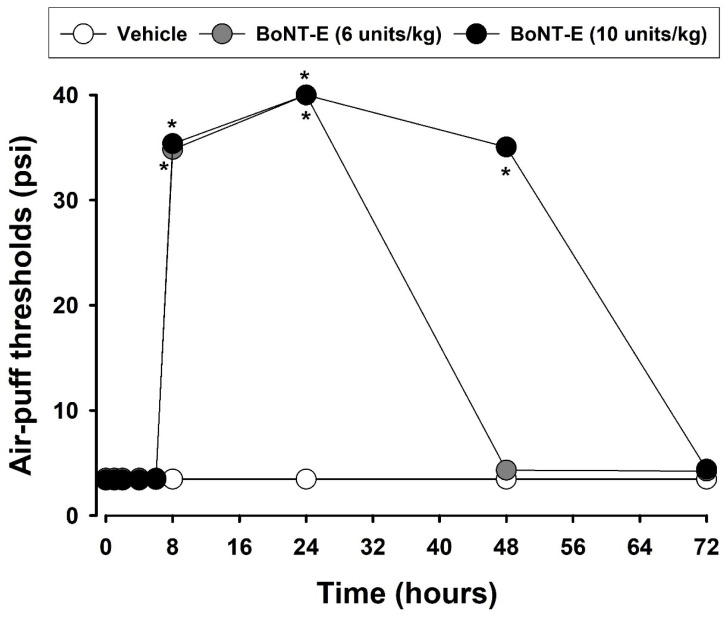
Effects of subcutaneously administered botulinum neurotoxin type E (BoNT-E) on mechanical allodynia in rats with trigeminal neuropathic pain. Vehicle treatment did not alter air-puff thresholds. However, BoNT-E treatment (6, 10 units/kg) on POD 3 significantly increased the air-puff thresholds compared to those of the vehicle-treated group, with anti-allodynic effects persisting for up to 48 h following a 10 units/kg injection of BoNT-E. Each group comprised seven animals. * *p* < 0.05, BoNT-E-treated vs. vehicle-treated group.

**Figure 5:**
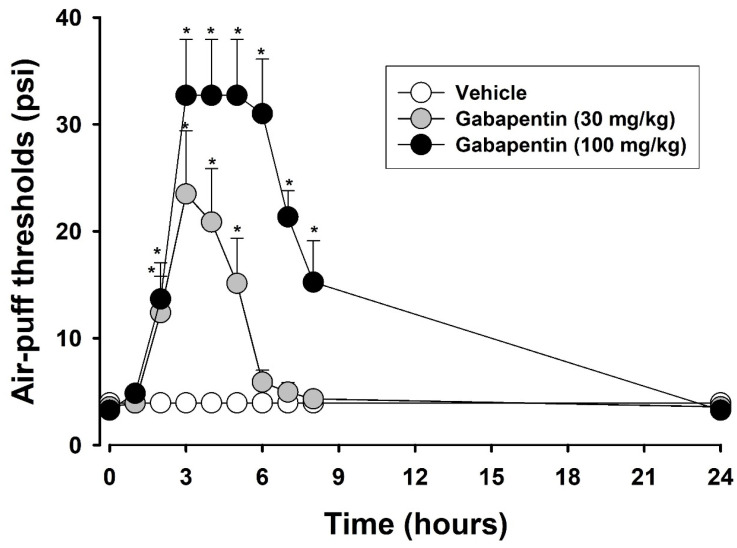
Effects of subcutaneously administered gabapentin on mechanical allodynia in rats with trigeminal neuropathic pain. Vehicle treatment did not alter air-puff thresholds. On POD 5, gabapentin treatment (30, 100 mg/kg) significantly alleviated mechanical allodynia compared to that of the vehicle-treated group, with the anti-allodynic effects lasting up to 8 h after a 100 mg/kg injection of gabapentin. Each group comprised seven animals. * *p* < 0.05, gabapentin-treated vs. vehicle-treated group.

Figure 4 depicts the effects of BoNT-E on mechanical allodynia following an injury to the inferior alveolar nerve. This study confirmed that an inferior alveolar nerve injury caused significant neuropathic mechanical allodynia. On POD 3, the subcutaneous administration of BoNT-E (6, 10 units/kg) produced significant anti-allodynic effects (F_(2,16)_ = 57.937, *p* < 0.05). The effects became evident after 48 h; no further effects were observed. The duration should be less than 48 h following a 10 units/kg injection of BoNT-E. Figure 5 illustrates the antinociceptive effects of gabapentin in rats with an inferior alveolar nerve injury. On POD 5, intraperitoneally injected gabapentin at doses of 30 or 100 mg/kg significantly alleviated neuropathic mechanical allodynia compared to that of the vehicle-treated group (F_(2,18)_ = 173.2, *p* < 0.05). The anti-allodynic effects of the 100 mg/kg dose were sustained for over 6 h but diminished by 24 h post-injection.

### 2.5. Subcutaneously Injected BoNT-E-Attenuated c-fos Expression in the Trigeminal Subnucleus Caudalis

**Figure 6:**
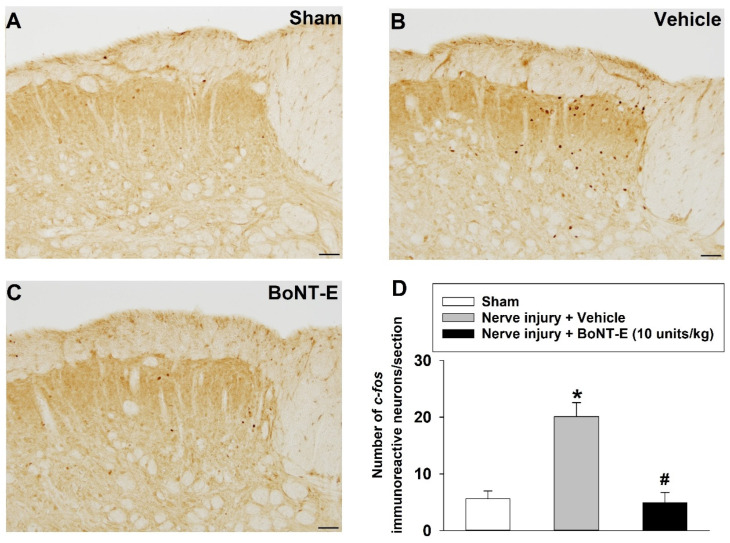
Effects of subcutaneously administered botulinum neurotoxin type E (BoNT-E) on *c-fos* expression. The *c-fos* immunostainings were conducted 5 days post-operation in the ipsilateral trigeminal subnucleus caudalis. (**A**) *c-Fos*-immunoreactive neurons in sham-operated rats. (**B**) *c-Fos*-immunoreactive neurons following vehicle treatment in rats with inferior alveolar nerve injury. (**C**) *c-Fos*-immunoreactive neurons following BoNT-E treatment (10 units/kg) in rats with inferior alveolar nerve injury. (**D**) Histograms illustrate the mean number of *c-fos*-immunostained neurons. Injury to the inferior alveolar nerve significantly upregulated the number of *c-fos*-immunoreactive neurons compared to that of the sham-operated rats, while BoNT-E treatment significantly reduced *c-fos*-immunopositive neurons compared to those of vehicle-treated rats. Each group comprised six animals. Scale bar: 50 μm. * *p* < 0.05, sham-treated vs. vehicle-treated group; ^#^ *p* < 0.05, vehicle-treated vs. BoNT-E-treated group.

Figure 6 illustrates the effects of subcutaneously administered BoNT-E on *c-fos* expression in rats with an inferior alveolar nerve injury. An injury to the inferior alveolar nerve resulted in a significant upregulated number of *c-fos*-immunoreactive neurons evoked by air-puff stimulation in the trigeminal subnucleus caudalis compared to that of the sham-treated group. Notably, most *c-fos*-positive neurons were localized in the superficial layers (laminae I–II) of the trigeminal subnucleus caudalis. The subcutaneous administration of BoNT-E at a dosage of 10 units/kg significantly decreased the number of *c-fos*-immunoreactive neurons evoked by air-puff stimulation in rats with an inferior alveolar nerve injury (*p* < 0.05).

## 3. Discussions

This study demonstrates the antinociceptive effects of BoNT-E. The subcutaneous administration of BoNT-E significantly reduced formalin-induced nociceptive behaviors and CFA-induced thermal hyperalgesia. Furthermore, BoNT-E effectively alleviated the neuropathic mechanical allodynia associated with an inferior alveolar nerve injury. Notably, BoNT-E treatment also suppressed *c-fos* expression in the trigeminal subnucleus caudalis, which was induced by air-puff stimulation in rats with an inferior alveolar nerve injury. Importantly, the analgesic efficacy of BoNT-E was of a longer duration than that of gabapentin. These findings highlight the potential of BoNT-E as a novel therapeutic approach to treating chronic pain conditions.

BoNTs are well-documented blockers of acetylcholine release at the neuromuscular junction, achieved through the cleavage of the SNARE complex [4,5]. BoNTs have gained prominence in both therapeutic and aesthetic applications, particularly in pain management by leveraging their neurotoxic properties. Previous experimental studies have suggested that BoNT-A might play a role in modulating chronic inflammatory pain. For instance, the subplantar administration of BoNT-A in a rat hind paw was shown to reduce carrageenan-induced mechanical hyperalgesia [10]. Additionally, subcutaneous BoNT-A injections inhibited the nociceptive behavior in rats with formalin [11] and CFA [12] treatment. These findings suggest that while BoNT-A primarily blocks acetylcholine release, it might also exert modulatory effects on chronic inflammatory pain. Recent clinical studies have begun to explore the potential role of BoNT-E in pain transmission. A single injection of BoNT-E significantly alleviated itch and pain symptoms in patients following excisions with linear repairs on the forehead, outperforming a placebo group [24]. These experimental results suggest that BoNT-E can control pain similarly to BoNT-A. However, experimental evidence on the efficacy of BoNT-E in chronic pain, including inflammatory pain, remains limited. The present study provides evidence that subcutaneously administered BoNT-E significantly inhibited formalin-induced nociceptive behaviors and CFA-induced thermal hyperalgesia. These findings suggest that BoNT-E holds promise as a therapeutic agent for managing chronic inflammatory pain.

Previous studies have also demonstrated the pivotal role of BoNT-A in modulating neuropathic pain. Specifically, peripherally administered BoNT-A has been shown to significantly reduce mechanical and thermal hypersensitivity following a partial sciatic nerve transection [13] and L5/6 spinal nerve ligation [14] in rats. Additionally, clinical studies have reported that peripheral BoNT-A administration significantly reduces mechanical allodynia in patients with neuropathic pain [15]. These findings underscore the therapeutic potential of BoNT-A as a promising candidate for developing novel treatments for neuropathic pain. However, there is limited experimental evidence regarding the effects of BoNT-E on neuropathic pain. The present study demonstrates that the subcutaneous administration of BoNT-E significantly inhibits neuropathic mechanical allodynia induced by an inferior alveolar nerve injury. These results indicate that BoNT-E might be a viable therapeutic option for managing neuropathic pain, highlighting its potential as a novel treatment approach for this condition.

In this study, we report for the first time that BoNT-E exhibits analgesic effects in various pain conditions. The concentration of BoNT-E used in this study was determined based on the experimental results of BoNT-A, which demonstrated an analgesic effect in previous experiments [16,17]. Although not presented in this study, it was noted that one unit of BoNT-E did not produce an analgesic effect. Consequently, the concentrations of 6 and 10 units used in this study were considered appropriate, as they exhibited analgesic effects in experimental animals. In the current study, the administration of 6 or 10 units of BoNT-E, which alleviates pain, did not result in any motor dysfunction when injected subcutaneously into the hind leg. Furthermore, no changes in body weight were observed over time. However, results from animal studies may not be directly applicable to humans, which represents a limitation of this type of research. Nevertheless, the findings of this study provide evidence suggesting that BoNT-E has an analgesic effect, and further clinical trials are necessary to validate this conclusion.

The present study demonstrated that the analgesic effect lasts for approximately 48 h following a single injection. These effects closely resemble the analgesic effect of BoNT-A, which has been documented in numerous studies [10,11,12,13,14,15]. Furthermore, this study explores the antinociceptive effects of BoNT-E by evaluating *c-fos* expression in the trigeminal subnucleus caudalis of rats with an inferior alveolar nerve injury. The injury led to a marked increase in *c-fos*-immunoreactive neurons within the trigeminal subnucleus caudalis. The subcutaneous administration of BoNT-E significantly attenuated the upregulation of *c-fos* expression triggered by air-puff stimulation. These results indicate that BoNT-E alleviates pain, as *c-fos* is commonly used as a marker for pain [25]. These findings align with the behavioral antinociceptive effects of BoNT-E, providing corroborative immunohistochemical evidence. Gabapentin remains the most commonly prescribed pharmacological agent for managing neuropathic pain [26]. In this study, the antinociceptive effects of intraperitoneally administered gabapentin (30, 100 mg/kg) were evaluated on POD 5 in rats with an inferior alveolar nerve injury. While gabapentin significantly alleviated neuropathic pain, its analgesic effects dissipated within 24 h post-administration. In contrast, the antinociceptive effects of BoNT-E persisted beyond 24 h. Since the results of this study were obtained from experimental animals, there are limitations in directly applying these findings to humans. Despite these limitations, these findings suggest that, compared to gabapentin, BoNT-E offers prolonged analgesic effects and holds significant promise as a novel therapeutic option for neuropathic pain management.

Previous studies have demonstrated that the administration of BoNT-E consistently mitigates the loss of pyramidal neurons in the hippocampus following focal brain ischemia, highlighting its neuroprotective effects through the inhibition of synaptic transmitter release [20]. Furthermore, BoNT-E has been shown to significantly decrease seizure incidence in a mouse model of mesial temporal lobe epilepsy by inhibiting specific morphological alterations, including the blockade of glutamate release and suppression of spike activity in pyramidal neurons [21,22]. These findings suggest that BoNT-E administration in the hippocampus might serve as a promising anti-seizure and antiepileptogenic intervention for epilepsy [23]. Despite these advancements, direct experimental evidence regarding the role of BoNT-E in pain management remains limited, and the fundamental mechanisms underlying its analgesic effects are largely unexplored. Structural studies using biophysical techniques, such as X-ray crystallography, have elucidated the receptor specificity of BoNT-E [3,27], supporting its ability to block vesicle fusion by cleaving SNAP-25 [18,19,28]. This raises the possibility that BoNT-E exerts its analgesic effect by modulating pain through a mechanism that inhibits SNAP-25. Previous studies have indicated that cultured rat sensory neurons exhibit resistance to the effects of BoNT-E, suggesting that the analgesic effects of BoNT-E may be mediated by mechanisms other than a direct action on sensory neurons [29]. These findings raise the possibility that BoNT-E-induced pain suppression does not primarily involve sensory neurons. In contrast, the present study demonstrated that the subcutaneous injection of BoNT-E did not cause motor dysfunction, as evidenced by the normal performance in the rotarod test. This lack of motor impairment suggests that BoNT-E was active but did not affect motor neurons under these conditions. Therefore, the discrepancies between studies are likely due to the differences in experimental methodologies, implying that alternative mechanisms may contribute to the analgesic effects of BoNT-E.

Recent studies have indicated that BoNT-A alleviates neuropathic mechanical allodynia by blocking voltage-dependent sodium channels [16] and mitigates trigeminal neuralgia by inhibiting the nucleotide-binding domain, leucine-rich repeat-containing protein (NLRP) 3-cytokine pathway within the trigeminal ganglion [17]. The analgesic effects of BoNT-A are believed to involve the inhibition of noncholinergic neurotransmitters, such as substance P, calcitonin gene-related peptide (CGRP), and glutamate, which play a role in neurogenic inflammation and sensitization [30,31,32]. Furthermore, a recent study demonstrated that BoNT-A is involved in neuroimmune pathways related to the pathogenesis of psoriasis by inhibiting the release of neuropeptides, including substance P and CGRP [33]. These experimental results suggest that multiple alternative mechanisms may contribute to the analgesic effects of BoNT-E. Therefore, further experimental studies are warranted to explore the underlying mechanisms of the antinociceptive effects of BoNT-E.

## 4. Conclusions

The subcutaneous administration of BoNT-E reduced formalin-induced pronociceptive behavior and CFA-induced thermal hyperalgesia. Additionally, BoNT-E alleviated the neuropathic mechanical allodynia and suppressed *c-fos* expression associated with an inferior alveolar nerve injury. Furthermore, the analgesic effect of BoNT-E was demonstrated to be sufficiently effective to replace the effects of gabapentin under the current experimental conditions. These findings suggest that BoNT-E holds significant potential as a therapeutic option for managing chronic pain in the future.

## 5. Materials and Methods

### 5.1. Animals

The present study used a total of 102 male Sprague Dawley rats weighing approximately 220–240 g. The rats were maintained under a controlled environment with a constant temperature, a 12 h light/dark cycle, and ad libitum access to food and water. All experimental procedures received approval from the Animal Care and Use Committee at Kyungpook National University (approval code: KNU 2023-0050) and adhered to the ethical guidelines for pain research in conscious animals established by the International Association for the Study of Pain [34]. Prior to the initiation of the experiment, the animals were acclimated to the testing environment for a minimum of 30 min, and all behavioral assessments were conducted between 09:00 and 18:00. The experiments and data analysis were conducted in a blinded manner. Blinding included distinguishing between control and drug-treated groups, conducting evaluations based on drug concentration, and analyzing the experimental results.

### 5.2. Animal Models of Orofacial Pain

Orofacial formalin responses: The formalin test was conducted to assess the inflammatory pain response in the facial region of the experimental rats [35,36]. In this procedure, a 5% formalin solution (30 µL) was subcutaneously administered into the vibrissa pad. Nociceptive behaviors, such as scratching or rubbing near the injection site, were monitored at 5 min intervals over a 60 min period. The orofacial formalin response displayed two distinct phases separated by an inactive period. The first phase (0–10 min) comprised an initial brief response, followed by an interval of reduced activity, leading to the second phase (10–60 min), characterized by sustained and prolonged nociceptive behavior [37,38].

CFA-induced thermal hyperalgesia: Chronic inflammation was induced by subcutaneously injecting 40 µL of CFA (Sigma–Aldrich, St. Louis, MO, USA) into the left vibrissa pad. To assess heat hyperalgesia, the experimental rats were acclimatized to a custom-designed acrylic restraint device (40–60 mm in height, 70–120 mm in length) equipped with an opening at the top to facilitate thermal stimulation and measure head withdrawal latency. The device was placed in a dark, soundproof room, and the animals were allowed to acclimate to the experimental conditions for at least 30 min before testing. After heat application, head withdrawal latency was recorded as previously described in the literature [39,40]. The application of thermal stimulation was conducted using an infrared thermal stimulator (infrared diode laser, LVI-808-10, LVI Tech, Seoul, Republic of Korea), calibrated to 11 W and 18.1 A. This intensity produced a latency of approximately 12 s when measured at a distance of 10 cm from the heat source to the vibrissa pad. Head withdrawal latency was recorded twice in 5 min intervals, with stimulation discontinued if latency exceeded 20 s to prevent tissue damage.

Trigeminal neuropathic pain: Animals were anesthetized using a combination of ketamine (40 mg/kg) and xylazine (4 mg/kg) solution. The left mandibular second molars were extracted under anesthesia, and mini-dental implants (1 mm in diameter, 4 mm in length; Megagen, Gyeongsan, Korea) were inserted to induce intentional damage to the inferior alveolar nerve [41,42]. A control group was established in which teeth were extracted without implant placement. Pain behavioral responses were evaluated using a series of 10 consecutive air-puff stimuli, each lasting 4 s, with a 10 s interval between stimuli, as previously described [41,43,44]. The parameters of the air-puff stimulus, including the pressure intensity, duration, and interval, were precisely controlled using a Pico-Injector (Harvard Apparatus, Holliston, MA, USA). The air puff was directed at specific facial areas, including the lower jaw, corners of the mouth, and whisker pad regions, through a 26-gauge metal tube (10 cm in length) positioned 1 cm from the face at a 90° angle. This setup allowed for the accurate identification of the most sensitive sites. Mechanical allodynia was assessed based on animals’ painful behavioral responses to 50% of the air-puff stimuli. The cut-off pressure for the air stimulus was set at 40 psi, consistent with previous studies [45,46]. Notably, naïve rats did not exhibit escape responses at pressures below this threshold. Only data from rats confirmed to have inferior alveolar nerve damage due to improper implant placement were included in the final analysis.

### 5.3. Immunohistochemical Staining of c-fos

On POD 5, animals received air-puff stimulation at an intensity of 40 psi in 10 s on/off intervals for 10 min. Three hours after the air-puff stimulation, rats were anesthetized using a consistent anesthetic protocol and subsequently underwent transcardial perfusion with normal saline, followed by 4% paraformaldehyde in 0.1 M phosphate buffer (PB, pH 7.4). A block of the caudal medulla area was dissected and subsequently post-fixed in the same solution for a duration of 2 h at a temperature of 4 °C. The tissue specimens were immersed overnight at 4 °C in 30% sucrose solution prepared in 0.1 M PB. Immunohistochemical staining was performed using the diaminobenzidine (DAB) method [46]. Tissue sections were cut into 30 μm slices using a freezing microtome (SM2000R, Leica, Wetzlar, Germany). The series of free-floating sections were rinsed in phosphate-buffered saline (PBS) and treated with 2.5% normal horse serum (Vector Laboratories, Newark, CA, USA) for a duration of 2 h. The tissue sections were incubated overnight at 4 °C with a rabbit polyclonal anti-*c-fos* antibody (1:1000; Abcam, Cambridge, MA, USA) and peroxidase-conjugated anti-rabbit immunoglobulin G (Vector Laboratories) for 2 h at room temperature. Following extensive rinsing with PBS, the tissue sections were further incubated in a buffer solution containing 3,3′-diaminobenzidine (DAB, Vector Laboratories) and hydrogen peroxide (pH 7.5, Vector Laboratories) for approximately one min. The stained sections were examined under BX-41 and U-RFL-T microscopes (Olympus, Tokyo, Japan). Neuronal profiles exhibiting *c-fos* immunopositivity were quantified in the superficial laminae (I–II) of the ipsilateral trigeminal subnucleus caudalis. These laminae are critical for processing pain within the spinal cord and the trigeminal spinal nucleus [47,48]. For statistical analyses, five consecutive sections containing immunolabeled neuronal profiles were selected from each subdivision of the ipsilateral trigeminal subnucleus caudalis.

### 5.4. Rotarod Test

Changes in motor performance following the subcutaneous administration of BoNT-E (10 units/kg) were assessed using a rotarod apparatus (Ugo Basile, Comerio, Italy), as previously described [37]. The rotarod was set to a constant speed of 16 rpm, with a maximum trial duration of 180 s. To ensure acclimatization, rats underwent two to three training sessions over two consecutive days prior to testing. Motor performance was evaluated over time, both before and after BoNT-E injection in the hind leg.

### 5.5. Chemicals

BoNT-E, at a concentration of 100 units/vial, was provided by JETEMA Co., Ltd. (Wonju, Republic of Korea) and reconstituted in 1 mL of sterile saline. The dosage per unit was 23 pg/units. In a nonclinical toxicity test conducted on rats, the approximate lethal dose was determined to be 3000 units/kg or higher for both males and females in a single-dose toxicity assessment (single administration). BoNT-E was injected using a 30 G insulin syringe. Gabapentin was procured from Tokyo Chemical Industry Co., Ltd. (Tokyo, Japan) and was dissolved in sterile saline. Formalin and CFA were procured from Sigma–Aldrich and similarly dissolved in sterile saline. In this experiment, the weight of the experimental animals was measured prior to administering the drug (BoNT-E and gabapentin), which was used at a specific concentration per kilogram of the animal’s body weight.

### 5.6. Experimental Protocols

Effects of BoNT-E on orofacial formalin responses: BoNT-E was administered subcutaneously at a dosage of 6 and 10 units/kg to the vibrissa pad region (*n* = 7 per group). Twenty-four hours later, a formalin solution (30 µL, 5%) was injected subcutaneously into the same region. This timing was chosen based on findings from the current study, which demonstrated that BoNT-E maintained antinociceptive effects at this time point. For each rat, the frequency of pronociceptive behaviors, such as scratching or rubbing the facial area near the formalin injection site, was continuously monitored over a 60 min in consecutive 5 min intervals.

Effects of BoNT-E on CFA-induced thermal hyperalgesia: In a previous study, the subcutaneous administration of CFA induced thermal hyperalgesia within 1 day after injection, peaking at 3 days and returning to baseline pre-operative levels by 14 days [49]. Therefore, in the current study, BoNT-E was administered subcutaneously at doses of 6 and 10 units/kg 3 days after CFA treatment. Thermal hyperalgesia was assessed by measuring changes in head withdrawal latency at 0, 2, 4, 6, 8, 10, 24, 48, and 72 h following the subcutaneous injection of BoNT-E or the vehicle (*n* = 7 per group).

Effects of BoNT-E on trigeminal neuropathic pain: The current study demonstrated that injury to the inferior alveolar nerve resulted in significant neuropathic mechanical allodynia. On POD 3, BoNT-E (6, 10 units/kg) was administered subcutaneously into the region of highest sensitivity. Changes in air-puff thresholds were recorded at 0, 1, 2, 4, 6, 8, 24, 48, and 72 h following the injection of BoNT-E or the vehicle (*n* = 7 per group). Furthermore, the antinociceptive effects of gabapentin were assessed in rats with an inferior alveolar nerve injury. Gabapentin was administered intraperitoneally at doses of 30 and 100 mg/kg on POD 5, and changes in air-puff thresholds were recorded at 0, 1, 2, 3, 4, 5, 6, 7, 8, and 24 h post-injection of gabapentin or the vehicle control (*n* = 7 per group).

Effects of BoNT-E on *c-fos* expression in the trigeminal subnucleus caudalis: On POD 3, BoNT-E was administered at a dosage of 10 units/kg to rats with an inferior alveolar nerve injury. Following the administration of BoNT-E or the vehicle (*n* = 6 per group), air-puff stimulation was applied at an intensity of 40 psi in 10 s on/off intervals for 10 min on POD 5. Three hours after the air-puff stimulation, *c-fos* immunoreactivity in the trigeminal subnucleus caudalis was evaluated.

### 5.7. Data Analysis

Changes in behavior data across groups were analyzed using repeated measures analysis of variance (ANOVA) followed by Holm–Sidak post hoc analysis. For multiple group comparisons, one-way ANOVA with Holm–Sidak post hoc analysis was conducted, while comparisons between two groups were assessed using Student’s *t*-test. A *p*-value of <0.05 was considered statistically significant for all analyses. Data are reported as mean ± standard error of the mean.

## Data Availability

The original contributions presented in this study are included in the article. Due to technical/time limitations, the raw data supporting the conclusions of this article will be made available by the authors on request.
